# Acute 5-HT7 receptor activation increases NMDA-evoked currents and differentially alters NMDA receptor subunit phosphorylation and trafficking in hippocampal neurons

**DOI:** 10.1186/1756-6606-6-24

**Published:** 2013-05-14

**Authors:** Maryam S Vasefi, Kai Yang, Jerry Li, Jeff S Kruk, John J Heikkila, Michael F Jackson, John F MacDonald, Michael A Beazely

**Affiliations:** 1 Department of Biology, 200 University Ave. W, Waterloo, ON, N2L 3G1, Canada; 2 Department of Physiology, University of Toronto, Medical Sciences Bldg., 1 King’s College Circle, Toronto, ON, M5S 1A8, Canada; 3 Robarts Research Institute, Western University, 100 Perth Drive, London, ON, N6A 5K8, Canada; 4 School of Pharmacy, 200 University Ave. W, Waterloo, ON, N2L 3G1, Canada

**Keywords:** 5-HT7, NMDA, Hippocampus, Isolated neurons, Phosphorylation, Trafficking

## Abstract

**Background:**

N-methyl-D-aspartate (NMDA) receptors are regulated by several G protein-coupled receptors (GPCRs) as well as receptor tyrosine kinases. Serotonin (5-HT) type 7 receptors are expressed throughout the brain including the thalamus and hippocampus. Long-term (2–24 h) activation of 5-HT7 receptors promotes the expression of neuroprotective growth factor receptors, including the platelet-derived growth factor (PDGF) β receptors which can protect neurons against NMDA-induced neurotoxicity.

**Results:**

In contrast to long-term activation of 5-HT7 receptors, acute (5 min) treatment of isolated hippocampal neurons with the 5-HT7 receptor agonist 5-carboxamidotryptamine (5-CT) enhances NMDA-evoked peak currents and this increase in peak currents is blocked by the 5-HT7 receptor antagonist, SB 269970. In hippocampal slices, acute 5-HT7 receptor activation increases NR1 NMDA receptor subunit phosphorylation and differentially alters the phosphorylation state of the NR2B and NR2A subunits. NMDA receptor subunit cell surface expression is also differentially altered by 5-HT7 receptor agonists: NR2B cell surface expression is decreased whereas NR1 and NR2A surface expression are not significantly altered.

**Conclusions:**

In contrast to the negative regulatory effects of long-term activation of 5-HT7 receptors on NMDA receptor signaling, acute activation of 5-HT7 receptors promotes NMDA receptor activity. These findings highlight the potential for temporally differential regulation of NMDA receptors by the 5-HT7 receptor.

## Background

N-methyl-D-aspartate (NMDA) receptors are tetrameric channels composed of two NR1 and two NR2 or NR3 subunits [1]. In the hippocampus, most NR2 subunits are either NR2A or NR2B [2] and there is evidence that heterotrimeric NMDA receptors containing both NR2A and NR2B are also present [3]. Several studies have examined the ability of serotonin (5-HT) receptors to modulate NMDA receptor activity. For example, in isolated cortical neurons, activation of 5-HT1A receptors inhibits NMDA receptor currents [4] and 5-HT3 receptor activation reduces NMDA receptor currents in cortical slices [5]. In contrast, in *Xenopus* oocytes, 5-HT2 receptor activation increases NMDA receptor currents [6] and in prefrontal cortical slices, 5-HT2A/2C agonists enhance NMDA-evoked responses [7].

Although first identified in the suprachiasmatic nucleus, 5-HT7 receptors are expressed throughout the CNS, including the hippocampus [8]. The effect of 5-HT7 receptor ligands on NMDA-evoked currents remains unknown however recent studies provide clear evidence for the regulation of glutamatergic signaling by 5-HT7 receptors. 5-HT7 receptors inhibit NMDA-induced neurotransmitter release in the dorsal raphe nucleus (DRN) and the physiological role of 5-HT7 receptors in circadian rhythms is associated with an inhibition of glutamate-dependent events [9,10]. In the suprachiasmatic nucleus, glutamate excitatory post-synaptic potentials (EPSPs) and glutamate-induced intracellular calcium levels are both inhibited by 5-HT7 receptor activation [11,12]. Taken together, these studies suggest that 5-HT7 receptor activation decreases NMDA and/or glutamate receptor signaling. In contrast, compared to wild-type, 5-HT7 receptor knock-out mice display a reduced induction of long-term potentiation (LTP), magnitude of LTP, and hippocampus-associated learning [13]. Therefore, although there is evidence that 5-HT7 receptors negatively regulate NMDA/glutamate signaling, deletion of 5-HT7 receptors decreased the magnitude of NMDA receptor-dependent events such as LTP.

5-HT7 receptors are Gα_s_–coupled, although they may couple to additional Gα isoforms including Gα_12_[14,15]. Recently we identified the 5-HT7 receptor as a regulator of platelet-derived growth factor (PDGF) β receptor expression and activity [16]. Activation of PDGFβ receptors by PDGF-BB selectively inhibits NR2B-containing NMDA receptor currents and this may be involved in the mechanism of PDGFβ receptor-mediated neuroprotection [17]. Intriguingly, 5-HT7 receptor-induced upregulation of the PDGFβ receptor was sufficient to protect neurons against NMDA-induced excitotoxicity [18]. Thus, we proposed that long-term activation of 5-HT7 receptors initiates pathways that ultimately negatively regulate NMDA receptor signaling.

To clarify the direct effects of 5-HT7 receptor activation on NMDA receptor signaling we examined the effects of 5-HT7 receptor agonists and antagonists on NMDA-evoked currents, NMDA receptor subunit phosphorylation, and subunit cell surface expression in the hippocampus. In isolated hippocampal neurons, application of the 5-HT7 receptor agonist, 5-CT, resulted in a rapid and sustained increase in peak NMDA-evoked currents. 5-HT7 receptor agonist treatment also differentially altered NMDA receptor subunit phosphorylation and cell surface expression. These data, along with our previous work, suggest a model for differential NMDA receptor regulation by 5-HT7 receptors over the short- and long-term.

## Results

**Figure 1:**
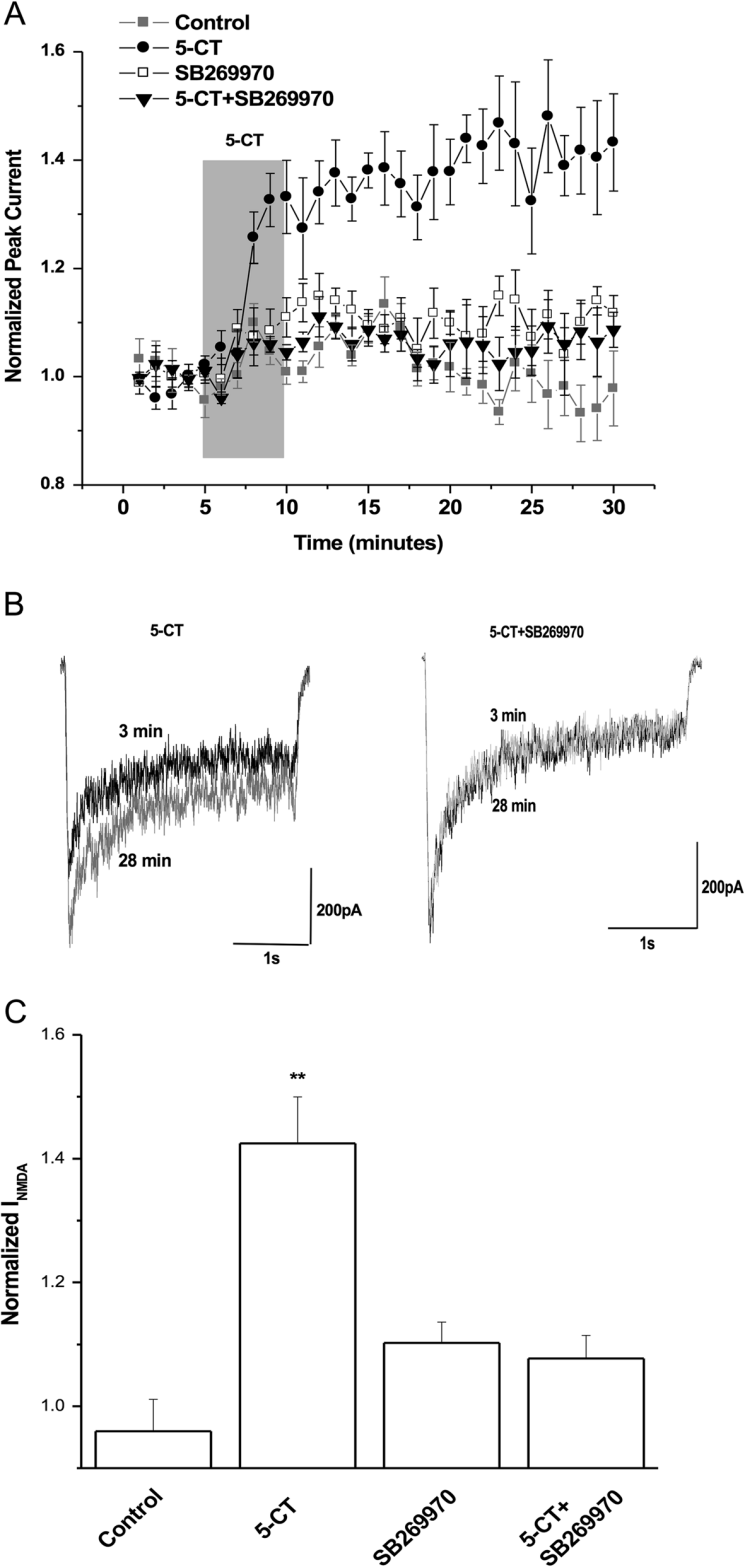
**5-HT7 receptor activation increases NMDA-evoked currents in isolated hippocampal neurons. A**) 50 nM 5-CT was applied to isolated CA1 hippocampal neurons for 5 min beginning at min 5 after a stable baseline of NMDA-evoked currents was established (closed circles). For the control data, vehicle (ECF) was continuously applied for 30 min. In samples treated with the 5-HT7 receptor antagonist, SB 269970 (1 μM) was included in the bath and was present before, during, and after 5-CT application. NMDA-evoked currents were recorded every 60 s by application of 50 μM NMDA, 0.5 μM glycine for 3 s. Recordings continued for at least 20 min after drug application. Peak currents were averaged for 5 min prior to applying 5-CT and were compared with currents averaged for values between 26 and 30 min. Data are representative of 5–6 independent experiments for each condition. **B**) Sample traces from the same cell treated with 50 nM 5-CT are shown at minute 3 (black trace) or minute 28 (gray trace). **C**) 5-HT7 antagonist SB269970 prevents the enhancement of I_NMDA_ by 5-CT. ** Indicates p < 0.01, one-way ANOVA (Turkey’s post hoc comparison).

Application of the 5-HT7 receptor agonist, 5-CT, to isolated hippocampal neurons for 5 min increased NMDA-evoked peak currents (Figure 1). NMDA receptor currents were evoked once every 1 min using a 3 s exposure to NMDA (50 μM) and glycine (0.5 μM). 5-CT was applied in the bath continuously for 5 min after a 5-min stable baseline recording. This application increased NMDA-evoked currents to 142.5 ± 7.4% (n = 5) compared with baseline, whereas NMDA-evoked currents in control cells were stable over the recording period (95.9 ± 5.2%, n = 6) (p < 0.01, one way ANOVA (Tukey’s post hoc comparison), Figure 1). The 5-CT-induced increase in NMDA-evoked currents was observed within minutes after 5-CT application and the increase in peak currents was sustained even after 5-CT was washed out (min 10 through 30). In addition to 5-HT7, 5-CT also activates 5-HT1 and 5-HT5 receptors [19,20]. However, the increase in NMDA-evoked currents by 5-CT was completely blocked by the 5-HT7 receptor-selective antagonist, SB 269970 (Figure 1).

**Figure 2:**
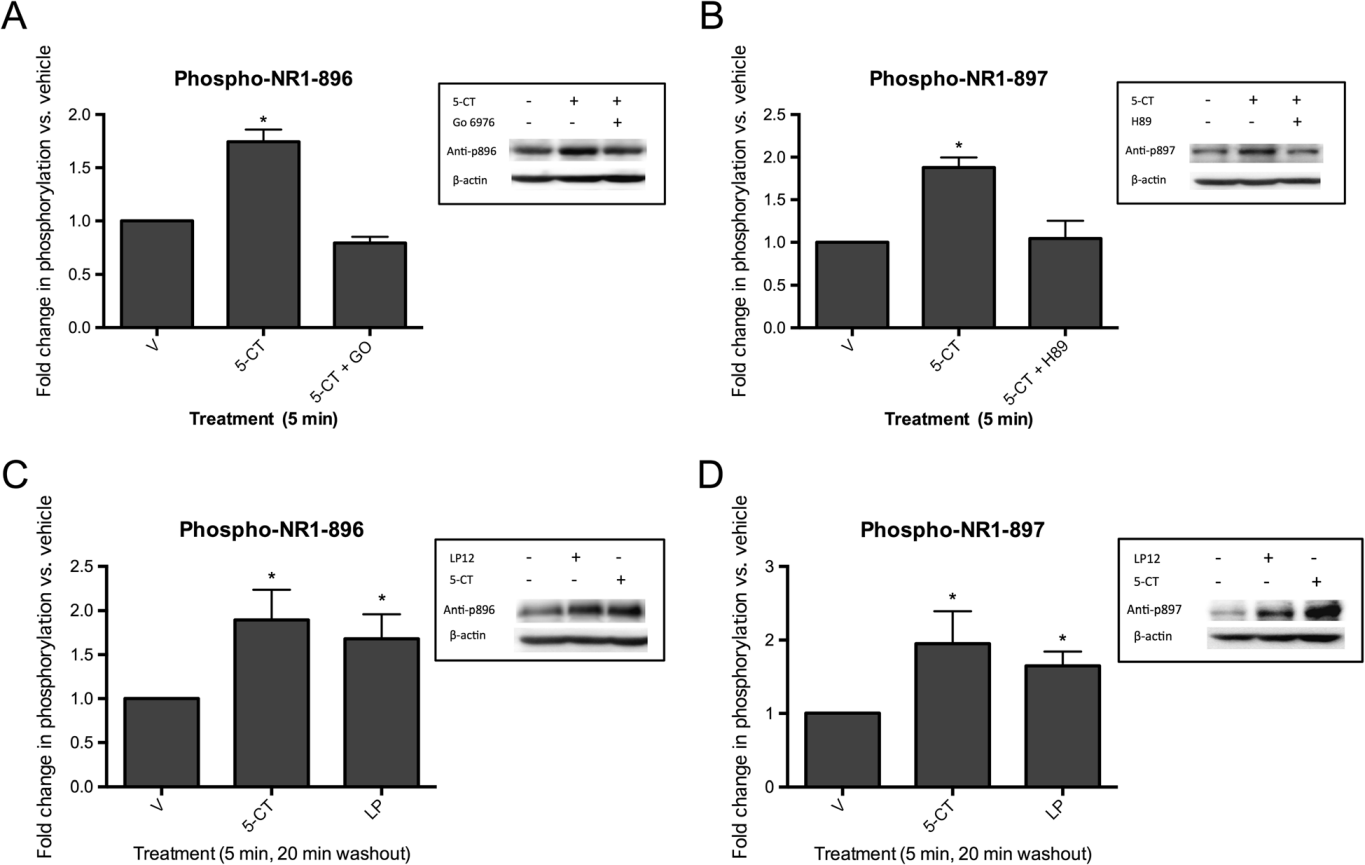
**5-CT treatment of hippocampal slices increases NR1 phosphorylation at serines 896 and 897. A**) Hippocampal slices were treated with 50 nM 5-CT in the absence or presence of 1 (micro)M Go 6983 (GO) for 5 min. Increases in the phosphorylation state at serine 896 were normalized to control (vehicle-treated slices) and to total NR1. Data represent the average and standard error of 10 independent experiments. * p < 0.5 compared to vehicle, ANOVA analysis with Dunnett’s post-test. INSET: representative Western blots showing serine 896 phosphoryaltion and the β-actin loading control. **B**) Hippocampal slices were treated with 50 nM 5-CT in the absence or presence of 10 μM H89 for 5 min. Data represent the average and standard error of 10 independent experiments. * p < 0.5 compared to vehicle, ANOVA analysis with Dunnett’s post-test. INSET: representative Western blots showing serine 897 phosphoryaltion and the β-actin loading control. **C**) Hippocampal slices were treated with 50 nM 5-CT or 300 nM LP 12 for 5 min, the slices were washed and incubated in drug-free buffer for an additional 20 min before lysis. Data represent the average and standard error of 8 independent experiments. * p < 0.5 compared to vehicle, ANOVA analysis with Dunnett’s post-test. INSET: representative Western blots showing serine 896 phosphoryaltion and the β-actin loading control. **D**) Slices were treated as in C. Data represent the average and standard error of 4 independent experiments. * p < 0.5 compared to vehicle, ANOVA analysis with Dunnett’s post-test. INSET: representative Western blots showing serine 897 phosphoryaltion and the β-actin loading control.

To determine if 5-HT7 receptor activation altered NMDA receptor subunit phosphorylation we incubated hippocampal slices with 50 nM 5-CT in the absence or presence of SB 269970. 5-HT7 receptors are coupled to Gαs in several cell lines [14,21-25] and the application of 5-CT to hippocampal slices robustly increased NR1 receptor phosphorylation at the PKA phosphorylation site, serine 897 (Table 1). 5-CT application also increased the phosphorylation of the NR1 subunit at PKC-site serine 896 (Table 1). The increase in the PKC-site serine 896 phosphorylation was blocked by the PKC inhibitor Go 6983 (Figure 2A) and the increase in the PKA-site serine 897 phosphorylation was blocked by the PKA inhibitor, H89 (Figure 2B). In Figure 1, a 5 min application of 5-CT resulted in a sustained elevation of NMDA-evoked currents for at least 20 min after washout. To determine if the observed changes in NR1 subunit phosphorylation were similarly sustained we treated hippocampal slices with 5-CT for 5 min, washed the slices, and waited an additional 20 min before homogenizing the tissue in lysis buffer. The phosphorylation state of both serines 896 and 897 remained significantly elevated above control for 20 min after the treatment with 5-CT (Figure 2C, D).

**Table 1:** 5-CT treatment differentially alters NMDA receptor subunit phosphorylation

**NMDAR subunit/phosphorylation site**	**Treatment (5 min)**		
	**5-CT**	**5-CT + SB**	**SB 269970**
NR1/896, n = 8	2.07 ± 0.39**	0.91 ± 0.31	1.06 ± 0.28
NR1/897, n = 8	1.51 ± 0.13**	1.00 ± 0.39	0.95 ± 0.11
NR2A/total, n = 10	0.71 ± 0.07**	0.97 ± 0.37	0.86 ± 0.17
NR2B/1472, n = 5	2.79 ± 0.37**	1.49 ± 0.61	2.86 ± 1.33
NR2B/1252, n = 10	1.39 ± 0.49	1.50 ± 0.23	1.53 ± 0.31
NR2B/1336, n = 7	1.25 ± 0.19	1.98 ± 0.31	2.36 ± 0.36**

Hippocampal slices were treated with 50 nM 5-CT in the absence or presence of 1 μM SB 269970 for 5 min. Changes in the phosphorylation state for each antibody were normalized to control (vehicle-treated slices) and to total NR subunit expression. For each experiment a β-actin antibody was used to confirm equal loading. Data presented are the average and standard error. * p < 0.5, ** p < 0.01, compared to vehicle.

**Figure 3:**
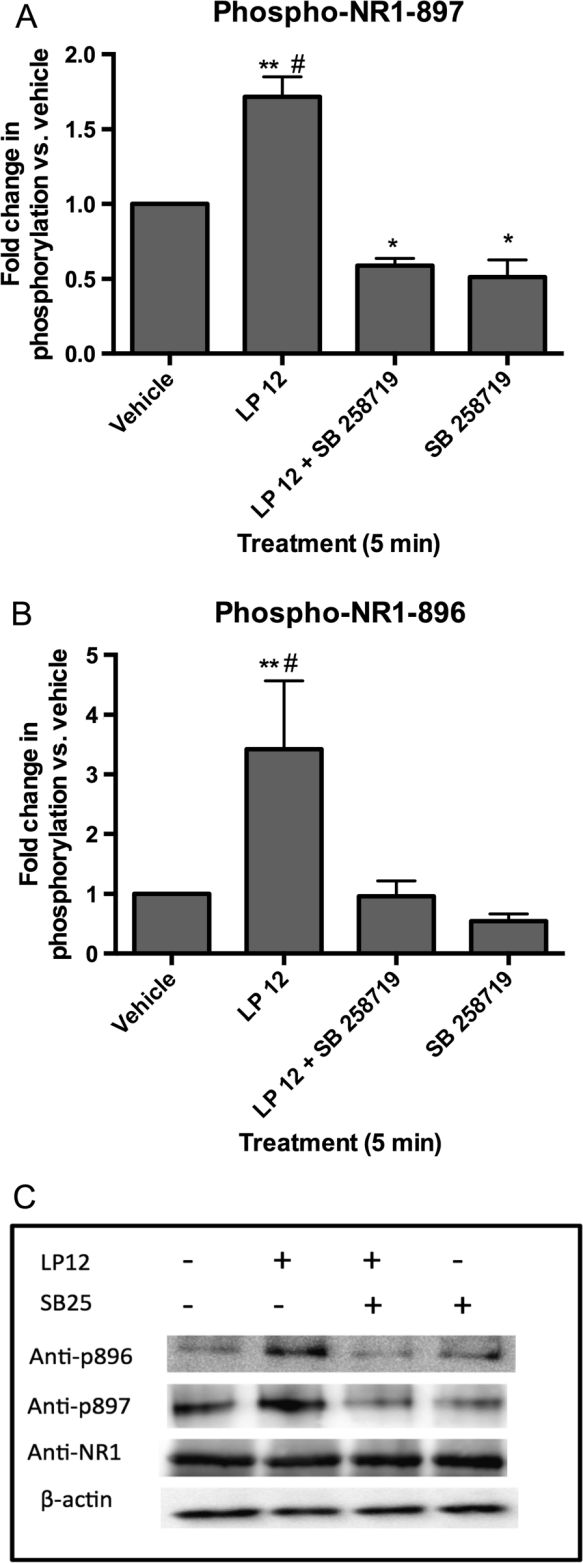
**5-HT7 receptor activation increases NR1 subunit phosphorylation. A**) Hippocampal slices were treated with 300 nM LP 12 in the absence or presence of 1 μM SB 258719 for 5 min. Increases in the phosphorylation state at serine 897 were normalized to control (vehicle-treated slices) and to total NR1. Data represent the average and standard error of 9 independent experiments. ** p < 0.01 LP 12 vs. vehicle, # p < 0.01 vs. LP 12 vs. LP 12 + SB 258719, * p < 0.5 compared to vehicle, ANOVA analysis with Bonferroni’s post-test. **B**) Hippocampal slices were treated as in A and western blots were performed using an anti-phospho-serine 896 antibody. Data represent the average and standard error of 10 independent experiments. ** p < 0.01 LP 12 vs. vehicle, # p < 0.01 vs. LP 12 vs. LP 12 + SB 258719, ANOVA analysis with Bonferroni’s post-test. **C**) Representative Western blots showing serine 896 and serine 897 phosphorylation, total NR1, and β-actin.

As mentioned above, 5-CT will also activate 5-HT1 and 5-HT5 receptors [19,20]. To confirm that the observed changes in NMDA receptor subunit phosphorylation were indeed due to the activation of the 5-HT7 receptor we incubated acutely-dissected hippocampal slices with the 5-HT7 receptor-selective agonist, LP 12 [26], in the absence or presence of the 5-HT7 receptor antagonists, SB 269970 and SB 258719 [27]. Similar to 5-CT, LP 12 increased NR1 receptor phosphorylation at the PKA phosphorylation site, serine 897 (Figure 3A, C) and this increased phosphorylation was sustained for at least 20 min (Figure 2D). Interestingly, we observed a larger increase in the phosphorylation of the adjacent, PKC-phosphorylated serine 896 (Figure 3B, C). However, the magnitude of the increase in serine 896 compared to serine 897 may be due to the lower basal phosphorylation state at serine 896. The increase in serine 896 phosphorylation was also observed for at least 20 minutes after LP 12 treatment (Figure 2C). The increased phosphorylation by LP 12 at both sites was blocked by the 5-HT7 receptor antagonists, SB 258719 (Figure 3) and SB 269970 (data not shown).

**Figure 4:**
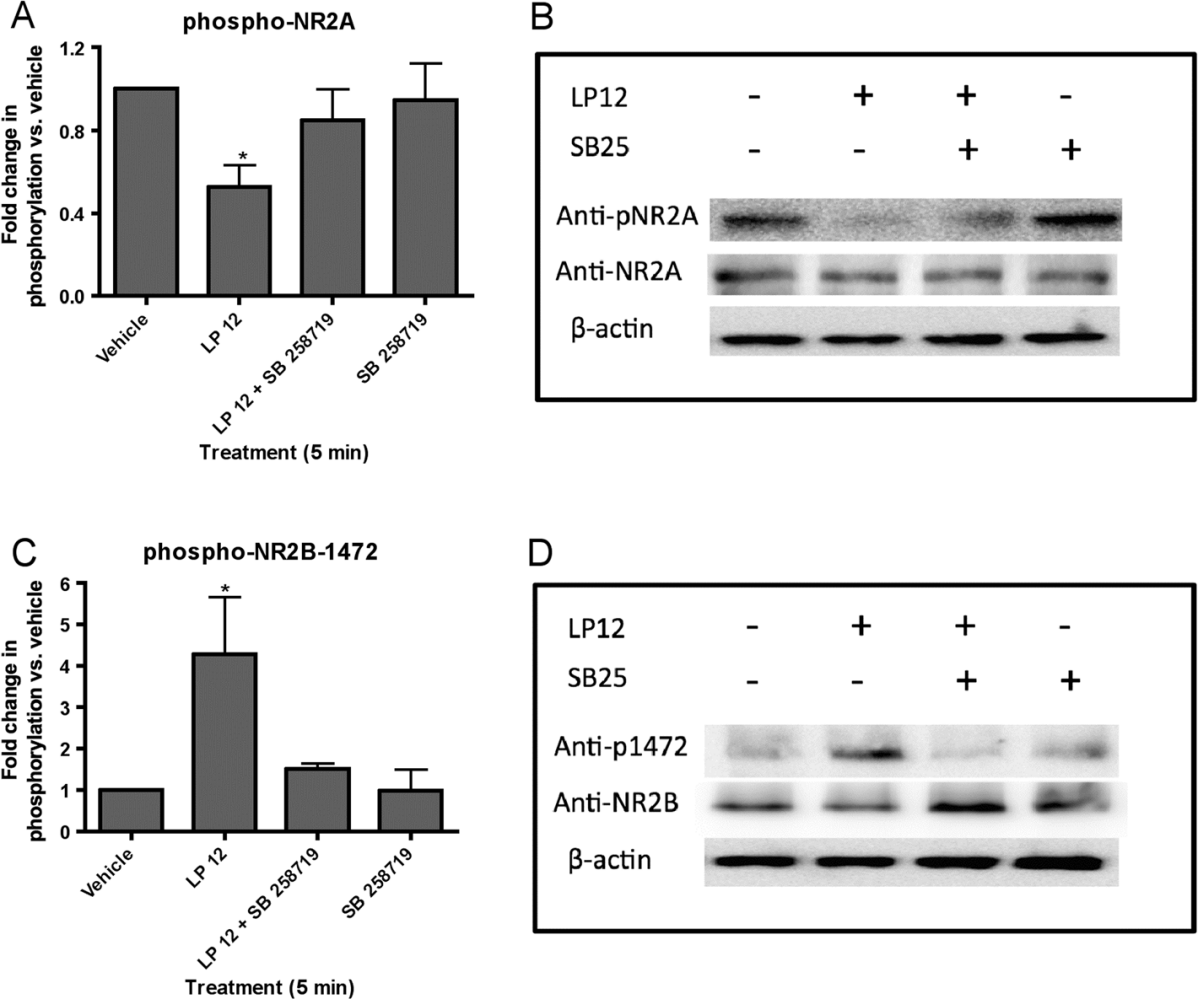
**5-HT7 receptor activation differentially alters NR2 subunit phosphorylation. A**) Hippocampal slices were treated with 300 nM LP 12 in the absence or presence of 1 μM SB 258719 for 5 min. Decreases in the phosphorylation state of NR2A were normalized to control (vehicle-treated slices) and to total NR2A. Data represent the average and standard error of 7 independent experiments. * p < 0.05 LP 12 vs. vehicle ANOVA analysis with Dunnett’s post-test. **B**) Representative Western blots using anti-phospho-pan-NR2A, NR2A, and β-actin. **C**) Hippocampal slices were treated as in A. Increases in the phosphorylation state of NR2B at tyrosine 1472 were normalized to control (vehicle-treated slices) and to total NR2B. Data represent the average and standard error of 6 independent experiments. * p < 0.05 LP 12 vs. vehicle ANOVA analysis with Dunnett’s post-test. **D**) Representative Western blots using anti-phospho-Y1472-NR2B, NR2B, and β-actin.

Application of LP 12 also differentially affected NR2 subunit phosphorylation. While LP 12 significantly reduced the phosphorylation state of the NR2A subunit (Figure 4A, B), LP 12 increased NR2B phosphorylation at tyrosine 1472 (Figure 4C, D). However the phosphorylation of NR2B at other tyrosine residues, 1252 and 1336, remained unchanged, with LP 12 inducing a 0.97 ± 0.12 (n = 12) and 0.93 ± 0.11 (n = 10) fold change, respectively, compared to control. The effects of LP 12 on the phosphorylation of NR2A and NR2B-Y1472 were both blocked by SB 258719 (Figure 4) and SB 269970 (data not shown). 5-CT treatment similarly enhanced NR2B-Y1472 phosphorylation and decreased NR2A phosphorylation (Table 1).

**Figure 5:**
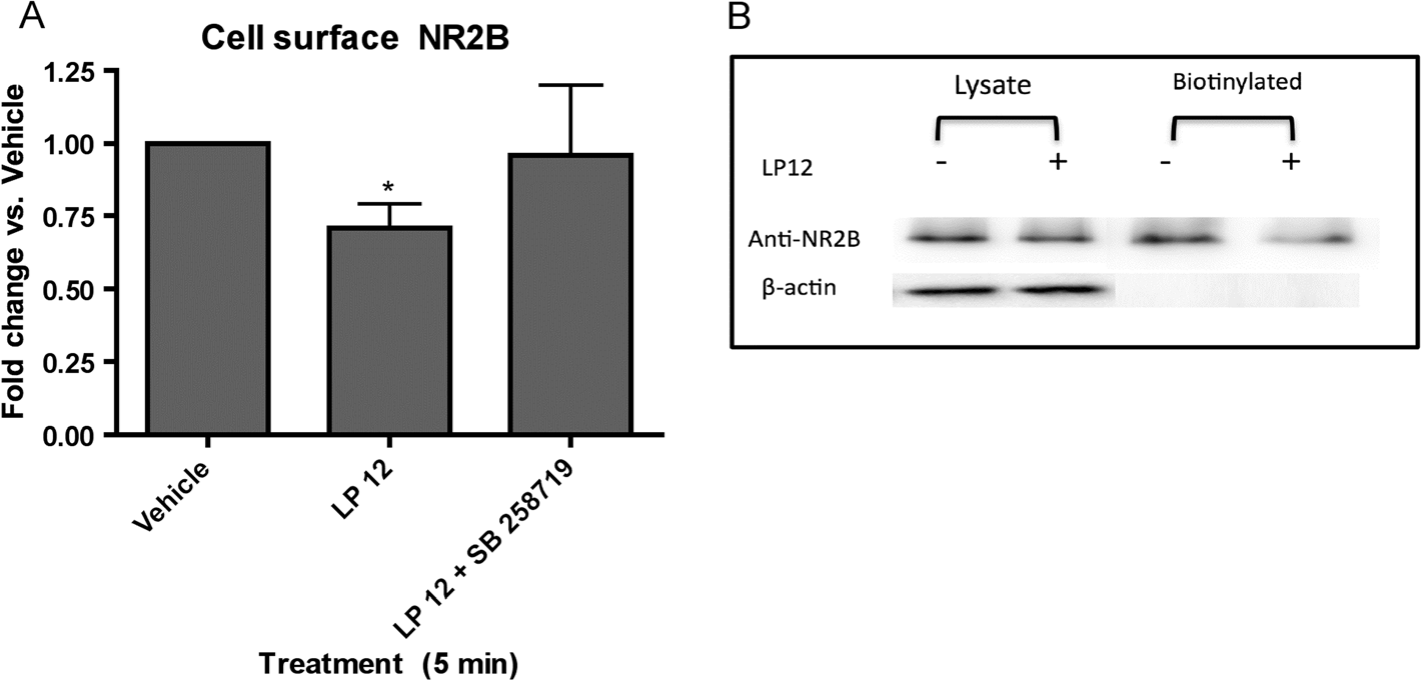
**LP 12 application results in the selective reduction of the surface expression of NR2B. A**) CA1 hippocampal slices were treated with vehicle, 300 nM LP 12, or LP 12 + 1 μM SB 258719 for 5 min. The slices were biotinylated and lysates were incubated overnight with streptavidin beads. Data represent the average and standard error of 6 independent experiments. * p < 0.05 LP 12 vs. vehicle ANOVA analysis with Dunnett’s post-test. **B**) Total lysates or biotinylated samples were blotted using antibodies against NR2B or β-actin.

Several receptors and signaling pathways regulate NMDA receptor subunit cell surface expression. To investigate the effects of 5-HT7 receptor agonist treatment on the cell surface expression of the NMDA receptors subunits we treated hippocampal slices with LP 12 in the absence or presence of SB 258719 for 5 min. In hippocampal slices LP 12 treatment did not significantly change the cell surface expression of NR1 (0.92 ± 0.13 vs. control) and NR2A (1.05 ± 0.13 vs. control), but NR2B cell surface expression was reduced by approximately 25% and this effect was blocked by SB 258719 (Figure 5).

## Discussion

**Figure 6:**
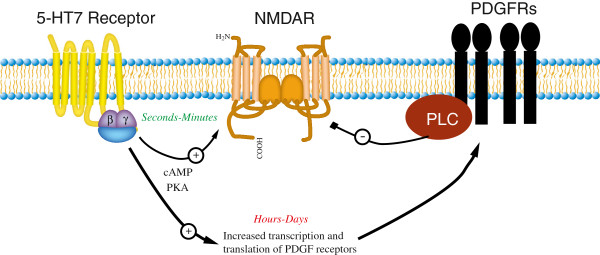
**Proposed model for NMDA receptor regulation by short- and long-term activation of 5-HT7 receptors.** Prolonged (2 to 24 h) activation of 5-HT7 receptors up regulates PDGF receptor activation and expression in hippocampal neurons, cortical neurons, and SH-SY5Y cells. The upregulation of PDGFβ receptors is sufficient to inhibit NMDA-induced neuronal cell death in primary cultures. In addition, NMDA receptor subunit expression is reduced. In contrast, acute (5 min) activation of 5-HT7 receptors increases NMDA-evoked currents and differentially regulates NMDA receptor subunit phosphorylation and cell surface expression. Figure modified from [18].

5-HT7 receptor knock-out mice display a reduced induction of LTP [13] and our finding that the acute 5-HT7 receptor activation increases NMDA-evoked currents may contribute to our understanding of how 5-HT7 receptors modulate synaptic plasticity. 5-HT7 receptor-mediated increases in NMDA-evoked currents may also contribute of our understanding of how 5-HT7 receptor activation potentiates bursting frequency in the CA3 hippocampal neurons [28] and increases CA1 extracellular population spike amplitude in CA1 hippocampal neurons [29]. Conversely, there is evidence that 5-HT7 receptor agonists decrease glutamate-induced intracellular calcium release [11] and the amplitude of glutamate EPSPs [12]. Based on the findings reported here and our previous report describing the ability of long-term 5-HT7 receptor agonist treatment to upregulate PDGFβ receptors [16,18], we propose that the activation of 5-HT7 receptors may regulate NMDA receptor activity via two temporally distinct pathways (Figure 6): Acute (5 min) activation of 5-HT7 receptors increases NMDA-evoked currents whereas long-term (2–24 h) activation of 5-HT7 receptors upregulates PDGFβ receptors, a receptor tyrosine kinase that inhibits NMDA receptor activity [17,18,30].

5-HT7 receptors are involved in aspects of learning and memory associated with hippocampal function [31]. For example, 5-HT7 receptor knock-out mice display impaired contextual fear-conditioning [13] and display a reduced ability to recognize new environments [32]. These and other studies have promoted interest in 5-HT7 receptors as a potential drug target in Alzheimer’s disease and are further supported by a study demonstrating an increase in memory formation by a 5-HT7 receptor agonist, AS 19 [33]. NMDA receptors are crucial components of learning and memory pathways in the hippocampus. Thus, the positive linkage between 5-HT7 receptors and NMDA receptor activity may explain how 5-HT7 receptors promote learning and memory as well as their involvement in LTP.

Since all three 5-HT7 receptor splice variants identified in rats are positively coupled with adenylate cyclase and display some level of constitutive activity [8], it is not surprising that the activation of 5-HT7 receptors in hippocampal slices increases the phosphorylation of the NR1 subunit at serine 897. On the NR2B subunit, tyrosine 1472 is required/involved in CaMKII binding and activation [34] and this phosphorylation site is linked to spinal pain transmission [35]. Interestingly, 5-HT7 receptors agonists promote pain after formalin injection in animal models [36] however others have found anti-nociceptive effects of 5-HT7 receptors [37,38]. These differences may be due to distinct central and peripheral pain pathways that both contain 5-HT7 receptors.

## Conclusions

In summary, we have now identified two pathways downstream of the 5-HT7 receptor that ultimately regulate NMDA receptor activity/signaling (Figure 6). We have demonstrated that the upregulation of PDGFβ receptors by long-term treatment with 5-HT7 receptor agonists is sufficient to protect hippocampal neurons against NMDA excitotoxicity [16,18] whereas acute activation of 5-HT7 receptors increased NMDA-evoked currents. These findings may help to explain why previous reports identified 5-HT7 receptors as both positive and negative regulators of NMDA receptor signaling.

## Materials and methods

### Reagents and antibodies

5-CT, LP 12 (4-(2-Diphenyl)-N-(1,2,3,4-tetrahydronaphthalen-1-yl)-1- piperazinehexanamide hydrochloride), and H89 were purchased from Sigma (St. Louis, MO, USA). The 5-HT7 receptor antagonists SB 258719 ((R)-3,N-Dimethyl-N-[1-methyl-3-(4-methylpiperidin-1-yl)propyl]benzene sulfonamide) and SB 269970 (R-3-(2-(2-(4-methylpiperidin-1-yl)ethyl)-pyrrolidine-1-sulfonyl)-phenol as well as Go 6983 were purchased from Tocris (Ellisville, MO, USA). Antibodies purchased from Millipore (Bellerica, MA) include anti-NR1, anti-phospho-896-NR1, anti-phospho-897-NR1, anti-NR2A, anti-phospho-pan-NR2A, anti-NR2B, anti-phospho-1252-NR2B, anti-phospho-1336-NR2B, and anti-phospho-1472-NR2B.

### Cell isolation and whole-cell recording

CA1 neurons were isolated from hippocampal slices of postnatal day 14–21 Wistar rats as previously described [39]. The extracellular solution was composed of 140 mM NaCl, 1.3 mM CaCl_2_, 25 mM N-2-hydroxyethylpiperazine-N’-ethanesulfonic acid (HEPES), 33 mM glucose, 5.4 mM KCl, and 0.5 μM tetrodotoxin, and 0.5 μM glycine, with pH of 7.3-7.4 and osmolarity ranging from 320–330 mOsm. Recordings were done at room temperature. The intracellular solution consisted of 11 mM ethyleneglycol-bis-(α-amino-ethyl ether) N,N’-tetra-acetic acid (EGTA) as intracellular calcium chelating buffer, 10 mM HEPES, 2 mM MgCl_2_, 2 mM tetraethyl ammonium chloride (TEA-Cl) to block K + channel, 1 mM CaCl_2_, 140 mM CsF, and 4 mM K_2_ATP. NMDA currents were evoked by rapid application of NMDA solution delivered from a multi-barreled fast perfusion system for 2 s every minute.

### Western blot

Hippocampal slices were prepared, treated with drugs for 5 min, and homogenized chilled lysis buffer (20 mM Tris–HCl at pH 7.5, 150 mM NaCl, 1 mM EDTA, 1 mM EGTA, 30 mM sodium pyrophosphate, 1 mM β-glycerophosphate, 1 mM sodium orthovanadate, 0.5% SDS, and 1% triton-X-100; supplemented with Halt Protease and Phosphatase Inhibitor (Thermo, Fisher, Markham, Ontario)) prior to use. Lysates were centrifuged at 14,000 × g for 20 min at 4°C and the supernatant was collected. The supernatant was subjected to SDS-PAGE and proteins were transferred to nitrocellulose membranes, blocked with 5% non-fat dry milk in Tris-buffered saline and 0.1% Tween for 1 h at room temperature or overnight at 4°C, and incubated in primary antibodies for 1 h at room temperature or overnight at 4°C. Membranes were washed three times in Tris-buffered saline with 0.1% Tween-20, incubated with HRP-conjugated secondary antibodies for 1 h at room temperature, washed again, and bound antibodies were visualized by the enhanced chemiluminescence using the chemiluminescent substrate (Millipore, Etobicoke, Ontario). Images of Western blots were taken on the Kodak 4000 MM Pro Imaging Station, and densitometric analyses were performed using the Kodak Molecular Imaging software. Membranes were then stripped and reprobed with other antibodies.

### Surface biotinylation assay

Hippocampal slices were incubated for 5 min with 5-HT7 receptor agonists and antagonists. Slices were washed in ice-cold ECF and incubated with 0.5 mg/ml Sulfo-NHS-LC-biotin (Pierce) for 30 min. The biotin reaction was quenched by washing with 10 mM glycine. Slices were washed twice more and homogenized in lysis buffer. Lysate protein concentrations were normalized and lysates were incubated with streptavidin beads overnight at 4°C (Sigma). Beads were washed three times in lysis buffer and boiled in loading buffer for 5 min before separation by SDS-PAGE.

### Statistical analysis

Statistical analysis of the data was performed using the Prism® GraphPad program. For electrophysiology, graphs and sample tracings were made using the Origin® program. Significance level was set at α = 0.05. Data was analyzed by one-way ANOVA or Student’s t-test where appropriate.

### Animals

All animal experiments were performed in agreement with the guidelines of the policies on the Use of Animals at the University of Waterloo (protocol #09-17) and the University of Western Ontario.

## Abbreviations

5-HT: 5-hydroxytryptamine; 5-CT: 5-carboxamidotryptamine; DRN: Ddorsal Raphe nucleus; EPSP: Excitatory post-synaptic potentials; LTP: Long-term potentiation; NMDA: N-methyl-D-aspartate; PDGF: Platelet-derived growth factor; SCN: Ssuprachiasmatic nucleus.

## Competing interests

The authors declare that they have no competing interests.

## Authors’ contributions

MSV carried out the Western blot and biotinylation experiments and KY performed the electrophysiology experiments under the supervision of JFM and MFJ. JL and JSK contributed to the biochemical experimentation and participated in the drafting of the manuscript. JJH co-supervised MSV and JSK and participated in the drafting the manuscript. MAB conceived of the study, participated in its design and coordination, participated in the drafting of the manuscript, and supervised JL, MSV, and JSK. All authors read and approved the final manuscript.
